# Bone formation by Irisin-Poly vinyl alchol modified bioglass ceramic beads in the rabbit model

**DOI:** 10.1007/s10856-024-06788-w

**Published:** 2024-03-25

**Authors:** Seong-Su Park, Ume Farwa, Hai-Doo Kim, Yong-Sik Kim, Byong-Taek Lee

**Affiliations:** 1 Department of regenerative medicine, College of Medicine, Soonchunhyang University Cheonan, Cheonan, Republic of Korea; 2 Institute of tissue regeneration, Soonchunhyang University Cheonan, Cheonan, Republic of Korea; 3 Department of Microbiology, College of Medicine, Soonchunhyang University Cheonan, Cheonan, Republic of Korea

**Keywords:** Bioglass, Irisin, Bone regeneration, Rabbit model, Polyvinyl alcohol

## Abstract

In the aging society, slow bone regeneration poses a serious hindrance to the quality of life. To deal with this problem, in this study, we have combined irisin with the bioglass regular beads to enhance the bone regeneration process. For this purpose, highly porous bioglass was obtained as spherical beads by using sodium alginate. The bioglass was evaluated by various analytical techniques such as SEM, EDS, XRD, and pore size distribution. The results depicted that porous bioglass was prepared correctly and SEM analysis showed a highly porous bioglass was formulated. On this bioglass, irisin was loaded with the assistance of polyvinyl alcohol (PVA) in three concentrations (50 ng/ml, 100 ng/ml, and 150 ng/ml per 1 g of bioglass). SEM analysis showed that pores are covered with PVA. The irisin release profile showed a sustained release over the time period of 7 days. In vitro, biocompatibility evaluation by the MC3T3E1 cells showed that prepared bioglass and irisin loaded bioglass (BGI50, BGI100, and BG150) are highly biocompatible. Alizarin Red staining analysis showed that after 2 weeks BGI50 samples showed highest calcium nodule formation. In vivo in the rabbit femur model was conducted for 1 and 2 months. BGI150 samples showed highest BV/TV ratio of 37.1 after 2 months. The histological data showed new bone formation surrounding the beads and with beads loaded with irisin. Immunohistochemistry using markers OPN, RUNX, COL, and ALP supported the osteogenic properties of the irisin-loaded bioglass beads. The results indicated that irisin-loaded bioglass displayed remarkable bone regeneration.

**Graphical Abstract:**

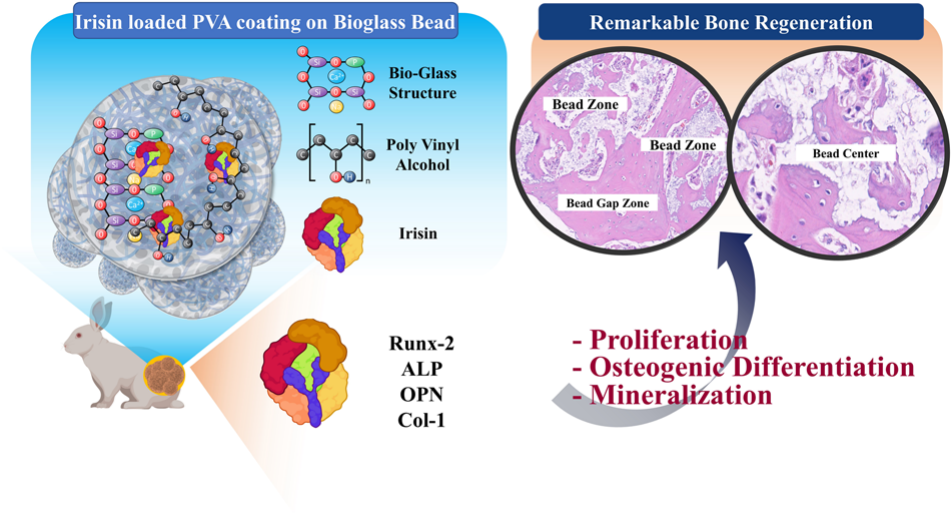

## Introduction

Human bone possesses a dynamic structure to facilitate multi-functionalities with remolding abilities that help the bone replace the micro-cracks and heal the damaged part. Although the human bone can regenerate, trauma due to bone injuries and defects results in severe illness [1, 2]. The critical-sized bone defect cannot heal through the innate bone regeneration. Bone regeneration ability in young individuals is particularly high [3, 4]. Especially when dealing with the aging society, the bone’s regenerative ability is insufficient to combat the consequences of bone injury [5]. To enhance the regeneration process of the bone, biomaterials such as hydroxyapatite and bioglass are an alluring facet. For biomaterial to meet desired outcomes, several merits are required.

The merits for the desired biocompatible biomaterials are very high; alternations in the strategies are required to meet these desired merits. Researchers are working to manipulate the desired outcome by modifying the biomaterials with various biomolecules that can elevate the innate regeneration ability and mechanical properties of the materials [6]. Researchers have reported that the combination of HAp to GO mechanical properties of coating materials can be improved [7]. The ultimate biomaterials should be biocompatible with the specific regenerative ability and, at the same time, should maintain the physical parameters such as degradability and strength [6, 8].

For bone regeneration, specific ions such as phosphate and calcium accelerate osteoblast differentiation, resulting in bone regeneration [9]. Such biomaterials which release these ions have attracted researchers to deal with bone-related problems. Bioglass (BG) is an exciting material as it releases ions such as silicon, phosphate, and calcium, which help activate the bone regenerative markers upon degradation. It exhibits high biocompatibility in both in vitro and in vivo [10–13]. Various metal ions (Ca, Si, Ag, Zn, and Cu) play important roles in the biomaterials such as imparting anti-bacterial properties as reported previously [14–18].

Incorporating various metal ions and hydrogels has been attempted to improve the bone regeneration ability of the bioglass. Hamed et al. [19] attempted to increase the bone regeneration ability by introducing Sr ions and incorporating the Sr-dopped bioglass in the hydrogel. The in vitro study suggested that Sr-dopped bioglass improved the bone regeneration ability of bioglass. Other studies report imparting metal ions such as Ag ions and Zr ions in the bioglass can improve the antibacterial properties of the bone regeneration materia [20, 21]. Mesoporous bioglass modified by the alginate suggested higher osteogenic ability by the in vitro analysis [22]. Previous research suggests that modification of the bioglass by various means can accelerate bone regeneration but the majority of the research has been conducted on in vitro models. To evaluate the osteoregenerative ability of the biomaterial, implantation in load-bearing defects such as the humerus, radius, and femur is essential [23].

With the progress of technologies, the quest to discover new biomolecules has led to the identification of a myokine known as irisin. Irisin has potential abilities that can accelerate osteoblast differentiation and proliferation. Physical activity is responsible for the release of irisin from the muscles [24–27]. After its release in the body, it participates in different regulatory responses such as weight loss and thermoregulation. Exploration has been focused on irisin to treat and prevent bone-related metabolic complications. One study reported that irisin knockout in mice delayed bone regeneration and decreased bone density [28]. A reversal experiment was performed to prove this theory by harvesting the MSCs from the knock-out mice and treating them with irisin. The results showed that the MSCs had developed osteogenic properties. Different studies have shown that irisin affects the physical structure of the bone [29, 30].

We loaded irisin on bioglass to evaluate the enhancement of the bone regeneration ability of irisin-loaded bioglass. Although, there have been attempts to modify bioglass by various means, but the effect of the bone regeneration ability has been studied by carrying out in vitro models. In this study, we aim to prepare an irisin loaded bioglass bead and evaluate its regeneration ability in the rabbit femur model which is a load bearing defect model. For this purpose, we prepared porous bioglass granules shaped into beads and loaded irisin on the beads using PVA as a binder. In vitro biocompatibility and calcium deposition were evaluated. We further assessed the regeneration ability by the in vivo evaluation of the rabbit femur model. The results demonstrate that irisin loaded bioglass displayed remarkable bone regeneration suggesting the use of this material for bone regeneration in future clinical applications.

## Material and methods

### Preparation of bioglass

To prepare the bioglass, a previously reported method was used [31]. Triethyl phosphate, tetraethyl orthosilicate, and poly(methyl methacrylate; PMMA) were obtained from Sigma-Aldrich. Nitrates of sodium and calcium were obtained from Daejung, South Korea. Following the reported method, all the reagents were mixed, and 60 min of stirring attained a solution that was clear. To acquire a gel consistency, the solution was stored at room temperature. For the drying process, the gel was treated at 70 °C for two days and 120 °C for one day. Next, milling was performed to obtain the product in powder form, which was further processed for 12 h at 700 °C to eliminate any residual materials, especially nitrates.

### Bioglass beads fabrication and characterization

Porous bioglass was acquired by mixing bioglass with PMMA at a ratio of 8:2 by stirring in the presence of 1.25% sodium alginate solution. A homogeneous solution was obtained as a result. A cross-linking solution of 10% CaCl_2_ was prepared. Using a 10 ml syringe, the slurry was dropped into the CaCl_2_ solution to obtain beads. The prepared beads were washed thoroughly with water and dried. The PMMA polymer and the beads were placed in the furnace for 2 h at 600 °C for calcination and burning. Sintering was performed at 950 °C for 4.5 h. SEM (scanning electron microscopy) was used for the morphological analysis, and the XRD was used to confirm the composition of the beads.

### Irisin loading

Human recombinant irisin was obtained from Regeron Inc. Chuncheon, South Korea. To load irisin on the bioglass beads a PVA solution was used (10% in water). Irisin of varying concentrations was added to the PVA solution to obtain samples with varying quantities of irisin, as mentioned in Table 1. The beads were added to the PVA solution containing varying quantities of irisin and stirred for 2 h. After 2 h, the samples were freeze-dried and labeled as BG, BGI50, BGI100, and BGI150 (Table 1).

**Table 1:** Irisin loading on BG beads

Sample name	Bioglass bead (w/v)	Irisin
BG	1 g	0 ng/mL
BGI50	1 g	50 ng/ml
BGI100	1 g	100 ng/ml
BGI150	1 g	150 ng/ml

### Compressive strength

A mechanical compression test was carried out to check the strength of the prepared samples by using a universal testing machine(AG-X, Shimadzu, Japan,) The crosshead speed of the test was set at 1 mm/min. For the compression test specimen dimension was 10 mm diameter and 5 mm hight.

### Irisin release

To determine the release profile of irisin from the beads, the samples were incubated in an appropriate amount of PBS for 7 days. Release was determined by using an ELSA kit following the manufacturer’s protocol. Each day, 500 μl of PBS was collected from the incubated samples to assess the release.

### Degradation rate

The degradation rate was determined by placing the samples in PBS solution at 37 °C in a shaking incubator. The samples were taken out of the solution at the predetermined time and placed in an oven for drying. Then, the weight of the samples was determined. The PBS solution was replaced every day. The following formula was used to determine the weight loss percentage.

1 $${\rm{Wl}} \% =(({\rm{Ws}}-{\rm{Wf}})/{\rm{Ws}})\times 100$$

Here, Wl is the weight loss, Ws is the starting weight, and Wf is the final weight. Meanwhile, the change in pH was also determined simultaneously using an pH meter (Thermo Scientific, USA).

### Alizarin red staining

Alizarin red staining was performed to evaluate the calcium deposition ability induced by the irisin-loaded bioglass beads. Pre-osteoblasts (MC3T3E1 cells) were used to assess the calcium deposition ability. Irisin-loaded beads were sterilized under UV for 90 min before use for in vitro evaluation. SPL hanging inserts were used to treat the cells with samples using DMEM media. Cells were treated with samples for 3 and 14 days with media change every third day. At the predestined time, cells were washed with PBS, fixed using PFA solution, and then treated with 2% ARS for 15 min at ambient temperature. Cells were observed under a light microscope after washing with DI water.

### Biocompatibility

The biocompatibility of the irisin-loaded beads was tested by treating samples with 3 × 10^3^ cells/well of the MC3T3E1 cell line. Typically, a 24 SPL hanging insert well plate was used. The biocompatibility was determined for 1, 3, and 7 days. The media contained 1% penicillin-streptomycin (Sigma-Aldrich), *α*-minimum essential medium, and 10% fetal bovine serum. The media was refreshed every 48 h. The cells were incubated at 37 °C under 5% CO_2_. At the predetermined time, the cells were washed with PBS and treated with 3-(4, 5-dimethylthiazol-2-yl)-25-diphenylterazoliumbromide (MTT, Sigma-Aldrich). Cells were incubated for 4 h after treatment with MTT. Dimethyl sulfoxide (Samchun Pure Chemical, Korea) was added, and a microplate reader was used.

Cell proliferation was performed following the same procedure. At the predetermined time, cells were washed with PBS and fixed with a 4% PFA solution. Following all the previously reported steps, cells were stained with Alexa 488 (cytoskeleton) and Hoechst 33342 (nuclei) (Invitrogen, USA). A fluorescence microscope (Olympus, FV10i-W, Tokyo, Japan) was used to visualize the samples.

### In vivo experiment

The rabbit femur model was used to evaluate the in vivo bone regeneration ability of the irisin-loaded beads. All animal ethics protocols provided by Soonchunhyang University’s ethical committee were followed (Approval number: SCH210042). Eighteen New Zealand White rabbits (2 weeks old) were obtained from Daehan Biolink Co, Limited (DBL). For the implantation of the samples, a surgical procedure was performed. The rabbits were anesthetized using isoflurane, and the incision site was shaved and sterilized with 70% ethanol and povidone. A trephine tunneling drill device made a 6 mm defect on the femur head after completing a 4 cm incision. The animals were divided into control, BG, and BGI50. The site of interest was harvested after 4 and 8 weeks, and the samples were stored in 10% formalin.

### Micro-CT

The extracted samples were observed for imaging under a 1076 μCT Scan device (Skyscan, UK). Seg3D software (Scientific Computing and Imaging Institute, USA) was used to contract the 3D images. Nrecon software version 1.6.9.8 was used to observe the new bone formation.

### Histology

The formalin-fixed samples were dehydrated using a series of ethanol and xylene to clarify any remaining alcohol. Samples were embedded in paraffin (Leica Biosystem, Germany) and sectioned (5 ± 2 µm thickness) using a microtome (Leica Biosystem, Germany). Tissue samples were stained by Hematoxylin and Eosin (H & E) and Masson’s Trichrome method. Immunohistochemistry was performed using DAB staining kit (3′-Diaminobenzidine, Agilent Technologies, US). The antibodies used were ALP (alkaline phosphatase), COL-1 (collagen-1), RUNX (runt-related transcription factor 2), and OPN (osteopontin) (Novus, USA). To quantify the expression of proteins, Image J software was used.

### Statistical analysis

All the experiments were performed thrice unless otherwise stated. Values are expressed as mean, standard deviation, and one-way ANOVA. Graphpad prism 8 was applied to determine the significant values. <0.05 p value was considered as significant.

## Results

### Bioglass characterization

**Fig. 1:**
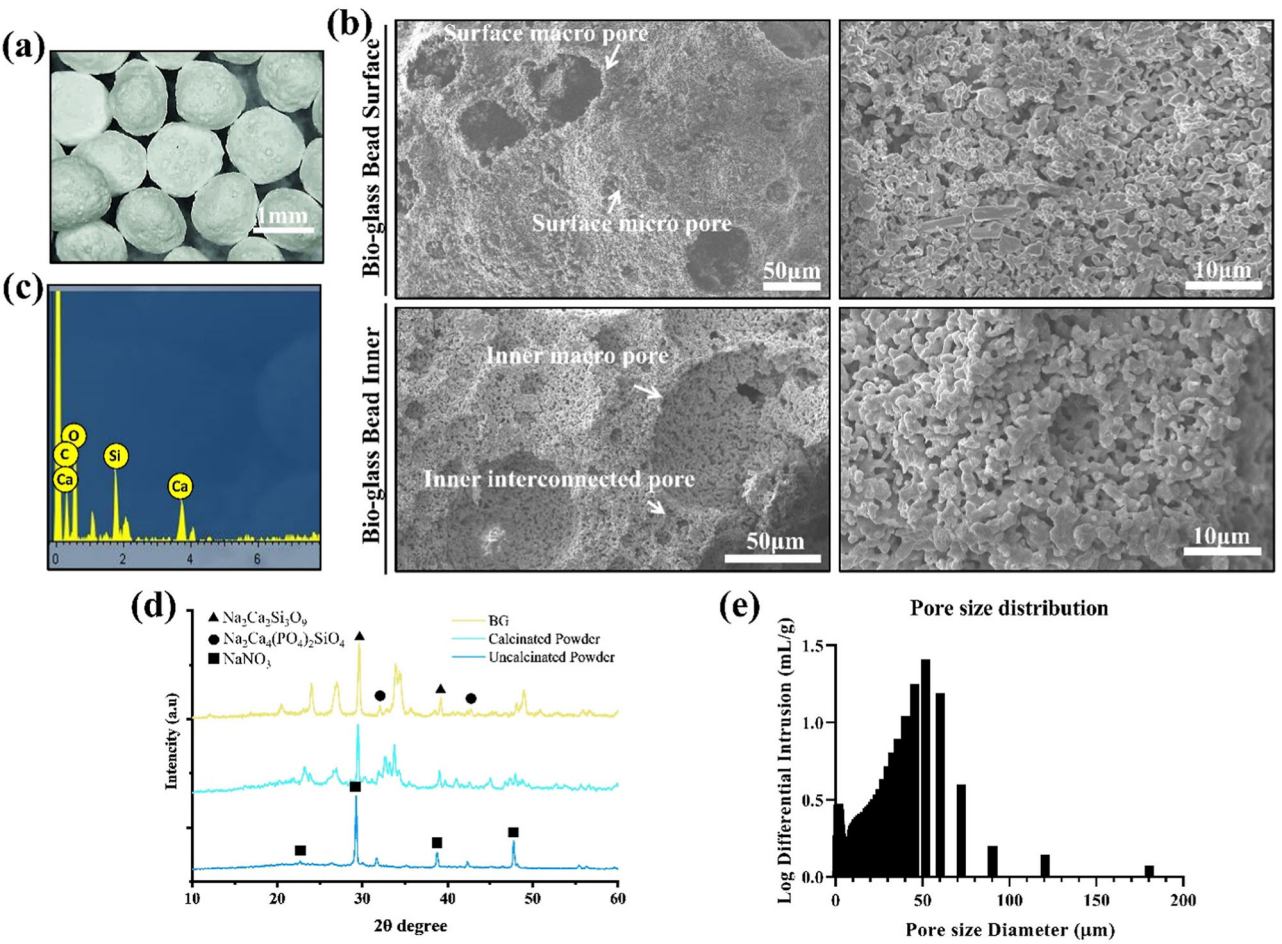
**a** Photograph showing bioglass beads, **b** SEM images of bioglass beads, **c** EDS of bioglass, **d** XRD of uncalcinated powder, calcinated powder, and bioglass, and **e** pore size distribution

Bioglass prepared by the sol-gel method was obtained in the form of small average-sized beads (1.8 mm, Fig. 1a). SEM images of the outer and inner morphology of the beads show the presence of the interconnected pores (Fig. 1b). The pores were generated in the beads due to the burning of PMMA. The EDS analysis reveals the elements constituting the bioglass (Fig. 1c). The pore size distribution shows that the average pores measured 50 μm (Fig. 1e). Figure 1d shows the XRD analysis. The analysis depicts the presence of bioglass without any other foreign phase. Also, it is revealed that the amorphous structures were removed after sintering. Although the treatment was done at a very high temperature (700 °C), Na_2_Ca_2_Si_3_O_9_ was still present, indicating a successful and pure production of the bioglass [32–34].

### Irisin loaded bioglass characterization

**Fig. 2:**
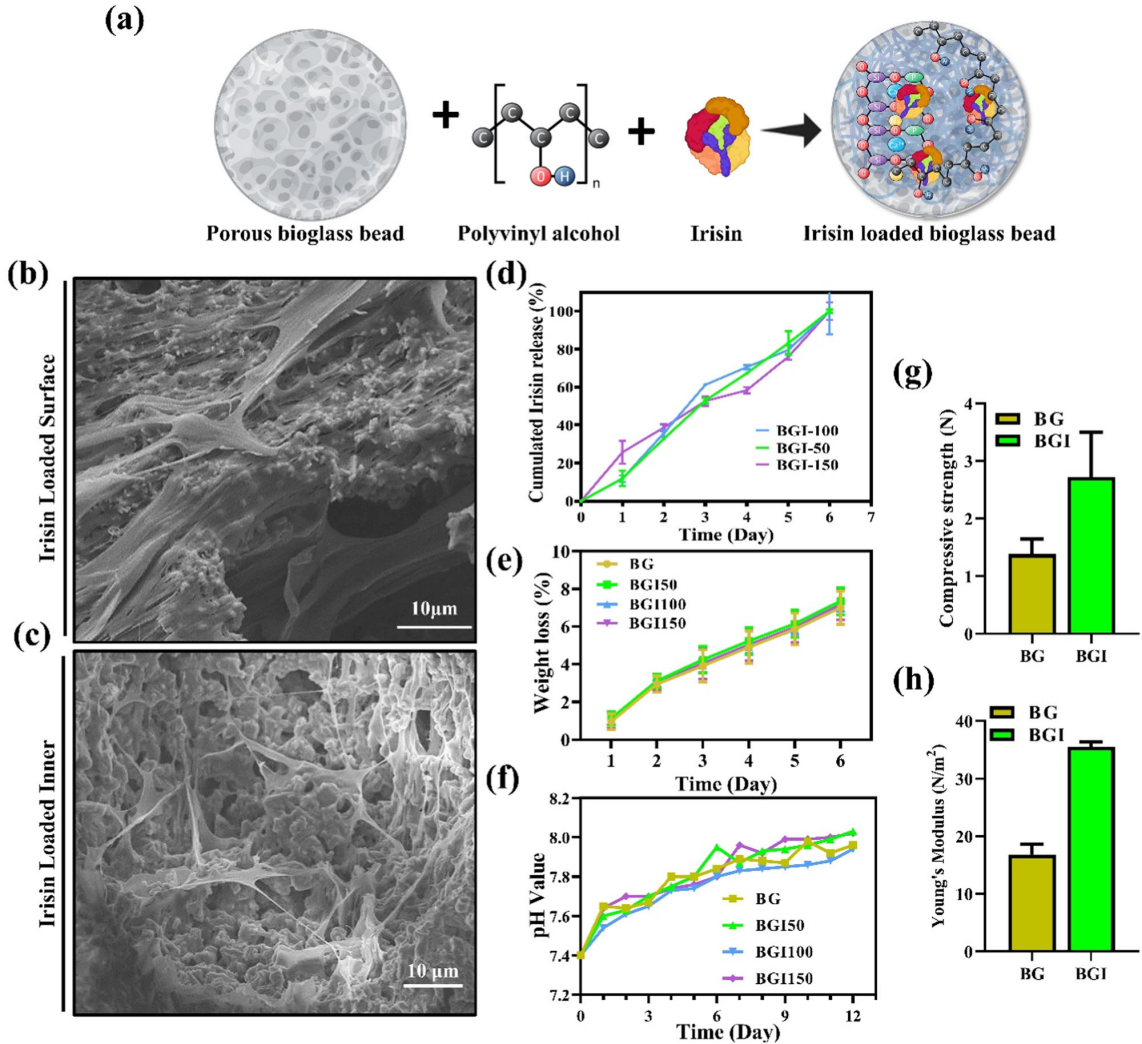
**a** Representation of bioglass, irisin, and PVA, **b** SEM of the surface of irisin and PVA loaded bioglass, **c** SEM of the inner cross-section of irisin and PVA loaded bioglass, **d** irisin release data, **e** degradation pattern of the samples, **f** changes in pH after immersion in SBF, **g** compressive strength, and **h** young’s modulus

The SEM analysis after loading the PVA and irisin shows shrinkage of the pores (Fig. 2b, c). It can be assumed that the shrinkage of pores can be due to the loading of the PVA and irisin. Irisin release assay was performed, as shown in Fig. 2d. The data showed that protein was released in a sustained manner within 1 week.

We evaluated the degradation ability of the bioglass and irisin-loaded bioglass samples. Within 7 days, 8% of degradation was displayed by the samples (Fig. 2e). Notably, the degradation rate was not affected by irisin and PVA loading. The degradation rate of the bioglass without irisin and with PVA and irisin was similar.

The compression strength and young modulus of the bioglass and irisin loaded bioglass were evaluated (Fig. 2g, h). After the modification of the bioglass the compression strength and young modulus were increased.

The bioglass and irisin-loaded bioglass samples showed an increase in the pH till 12 days (Fig. 2f). The samples showed an increase in pH to 8 when soaked in the stimulated body fluid for 12 days. When the bioglass samples are soaked in the stimulated body fluid, ions exchange between the solution and the bioglass samples, thereby increasing pH [35]. Previous studies have shown that alkaline pH is beneficial for bone regeneration [36].

### Alizarin red staining

**Fig. 3:**
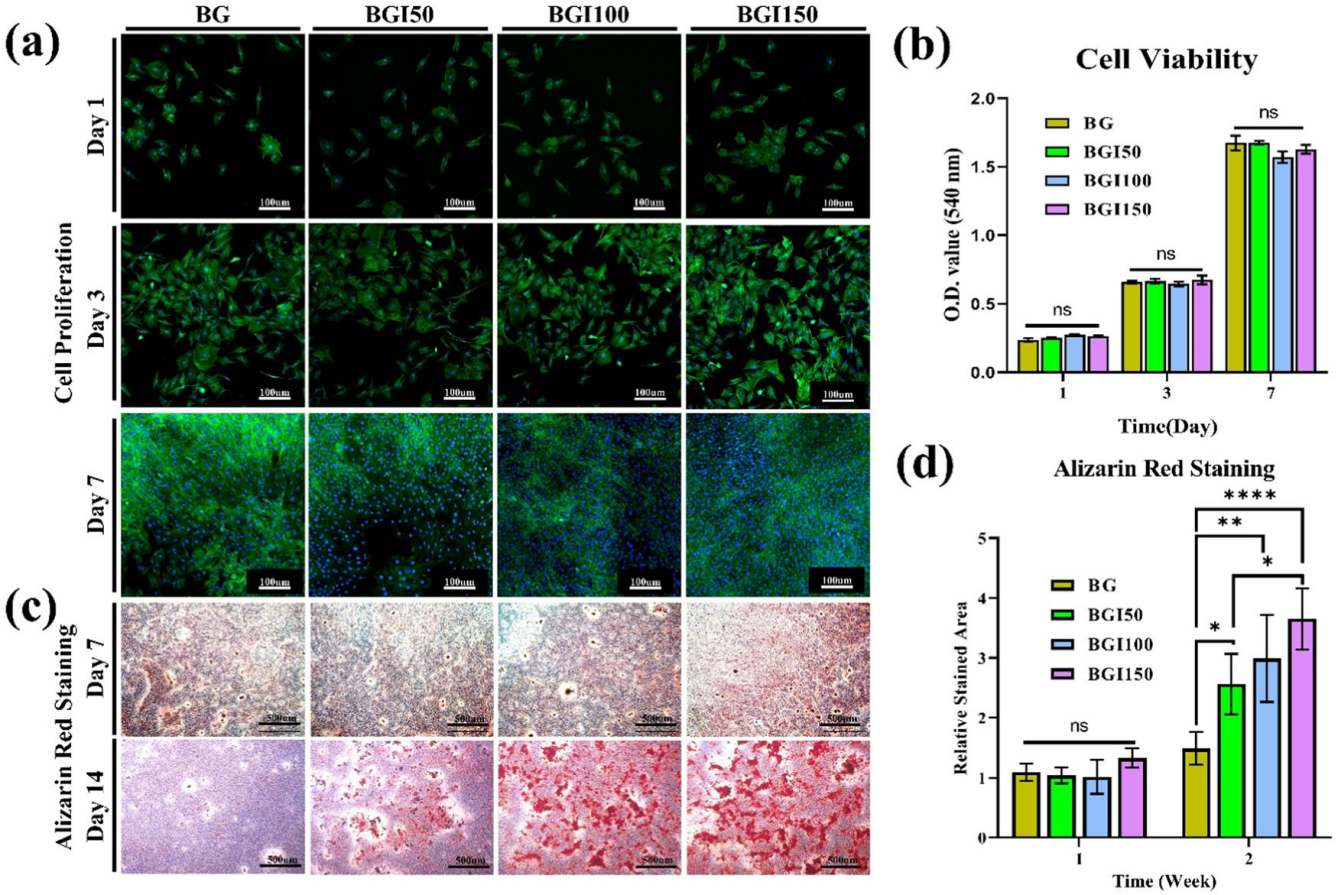
**a** Cell proliferation for 1, 3, and 7 days, **b** cell viability analysis, **c** alizarin red stained images, and **d** quantification of alizarin red staining. (****P < 0.0001, **P < 0.01, *P < 0.05,and ns not significant; *n* = 3)

To evaluate the Ca nodules formation ability of the samples, Alizarin Red staining was used (Fig. 3c, d). Bioglass and irisin-loaded bioglass samples were cultured with MC3T3E1 cells. Calcium nodules were observed after two weeks of seeding the cells. The quantification of the staining results showed that samples with the highest irisin concentration loaded on the bioglass BGI150 showed the highest extent of calcium nodule formation. Previously, PeiKai et al. reported that as compared to only bioglass, rhBMP9 protein loaded bioglass displayed higher Ca nodule formation as indicated by the Alizarin red staining [37].

### Biocompatabilty

To evaluate the biocompatibility of the bioglass beads and the irisin-loaded bioglass beads, cell proliferation of the MC3T3E1 cells was evaluated. Biomaterials for the hard tissue application such are widely evaluated by MC3T3E1cells as in vitro study model [38–40]. The quantitative evaluation was performed by the MTT test (Fig. 3b). The optical density showed that all the samples were highly biocompatible and increased with time. After 7 days, samples showed the highest optical density. Bioglass incorporated materials show high biocompatibility as reported previously [41]. The results assured that irisin loading on the bioglass had no harmful effect, and cell proliferation was remarkable (Fig. 3a). Confocal images were taken to determine cell proliferation. The images showed that cell proliferation was estimable, and as time passed, the rate of cell proliferation also increased.

### In vivo implantation

**Fig. 4:**
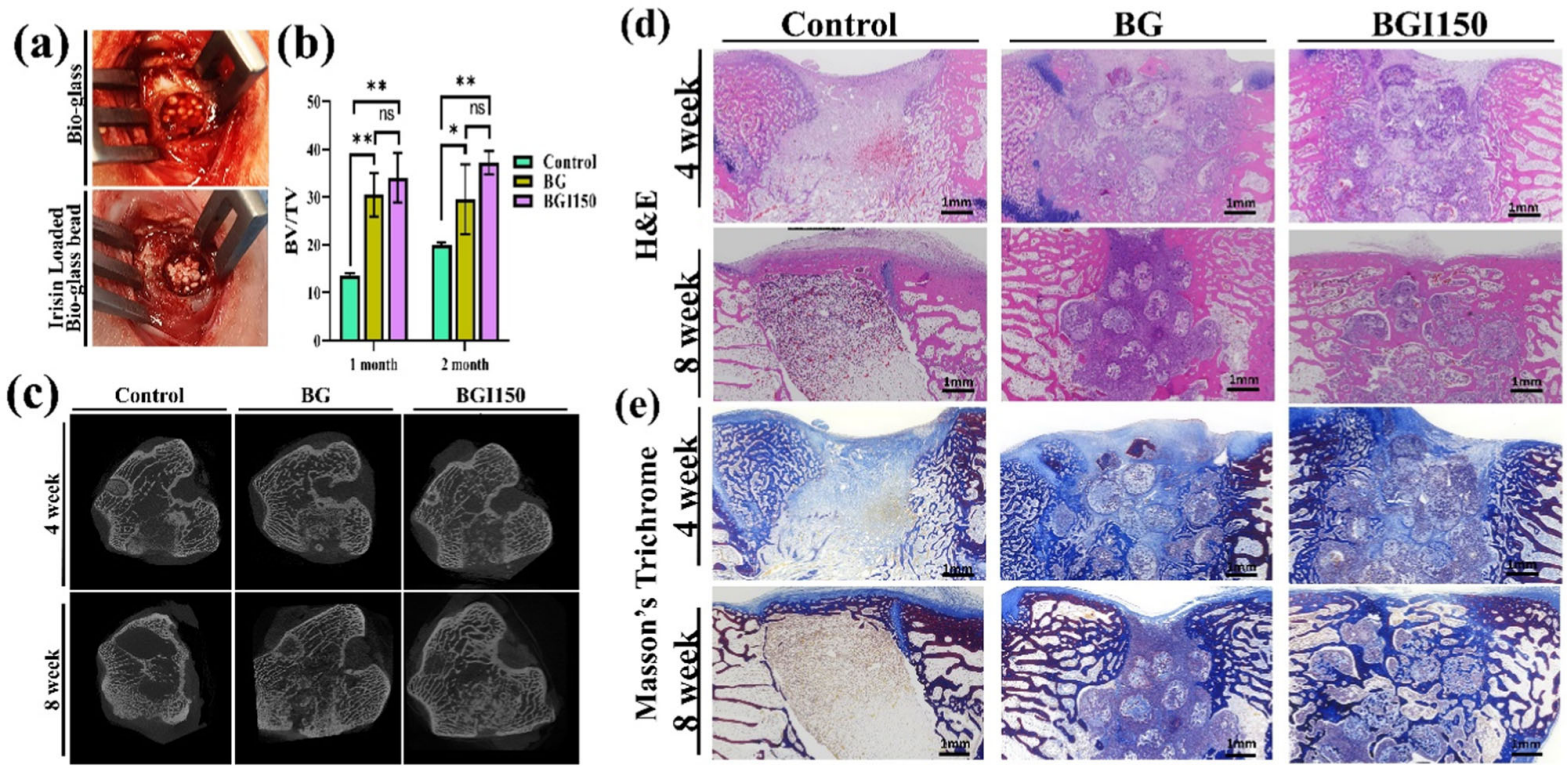
**a** In vivo implantation of the bioglass beads, **b** BV/TV ratio, and **c** micro-CT analysis images, **d** H&E staining images, and **e** Masson’s Trichrome staining images for 4 and 8 weeks. (**P < 0.01, and ns not significant; *n* = 3)

Figure 4a shows the in vivo implantation, and BG and BGI150 were selected regarding the control. The defect site without treatment served as the control. The samples were implanted for 4 and 8 weeks. Micro-CT analysis of the implanted samples is shown in Fig. 4c. The BV/TV ratio for the control, BG, and BGI150 was 13.6, 30.5, and 34.9 for 4 weeks (Fig. 4b). For 8 weeks, the ratio was 19.9, 29.5, and 37.1 for control, BG, and BGI150, respectively.

Figure 4d shows the histology data for the 4 and 8 weeks samples. In the control, there was no degraded bioglass. In the case of the BG and BGI150 samples, degraded bioglass was seen in the histology samples. Bone formation was observed in the BG and BGI150 samples after 4 weeks of implantation and was concentrated more toward the defect’s peripheral region than the center. The center was filled with a more bone-marrow-like structure. Notably, although the nature of the bioglass is degradable, there was no inflammation or immune response. This shows the highly biocompatible nature of the bioglass. A noticeable difference was observed between the bioglass and irisin-loaded bioglass in the two months’ samples. Bone formation was observed in the irisin-loaded bioglass sample. The degradation products of the bioglass were also observed in the 8 weeks samples.

We also performed the Masson-trichrome staining for the 4 and 8-week samples (Fig. 4e). MT staining showed collagen formation in the bioglass and irisin-loaded bioglass samples. Bioglass is known to induce collagen formation [42]. The similarity was seen in collagen formation between the bioglass and irisin-loaded samples, whereas enhanced bone formation was observed in the irisin-loaded samples. Histology analysis showed coherence with the micro-CT data.

**Fig. 5:**
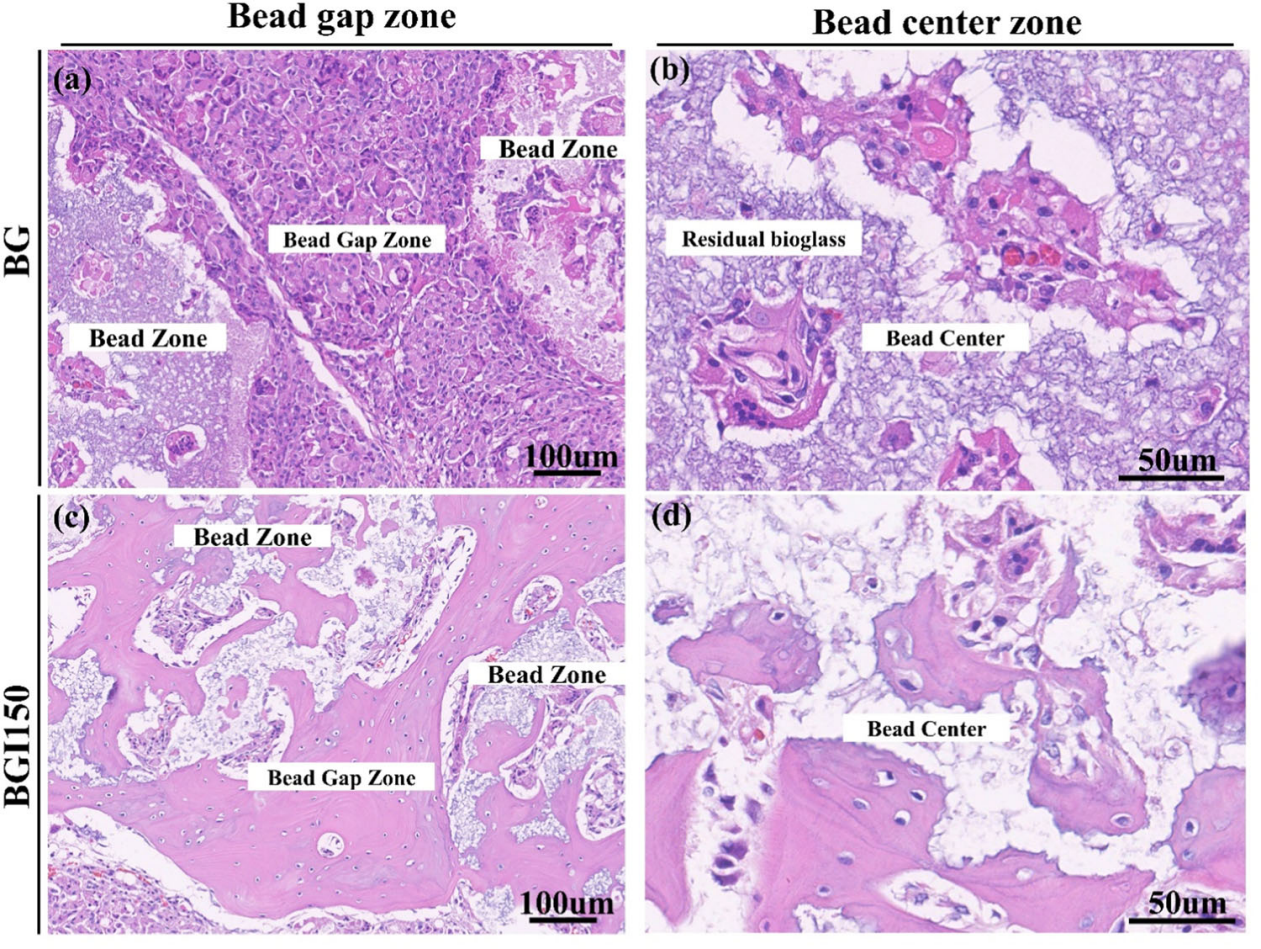
Magnified H&E staining images of **a** between the beads, **b** inside the beads of bioglass, **c** between the beads, and **d** inside the beads of irisin-loaded bioglass

Figure 5 shows the magnified view of the 8 weeks of bone formation for both bioglass and irisin loaded bioglass. In the case of the irisin-loaded bioglass, bone formation between the beads was observed after 8 weeks (Fig. 5c). Also, new bone formation was observed within the beads (Fig. 5d), whereas, in the only bioglass sample, a marrow-like structure was observed after 8 weeks (Fig. 5a, b). It was evident that irisin enhanced the bone formation ability of the bioglass. The presence of new bone in the bead center supports the fact stated earlier that porous bioglass facilitates cell migration inside the beads. This cell migration and new bone formation inside the bone are enhanced by the irisin loading.

**Fig. 6:**
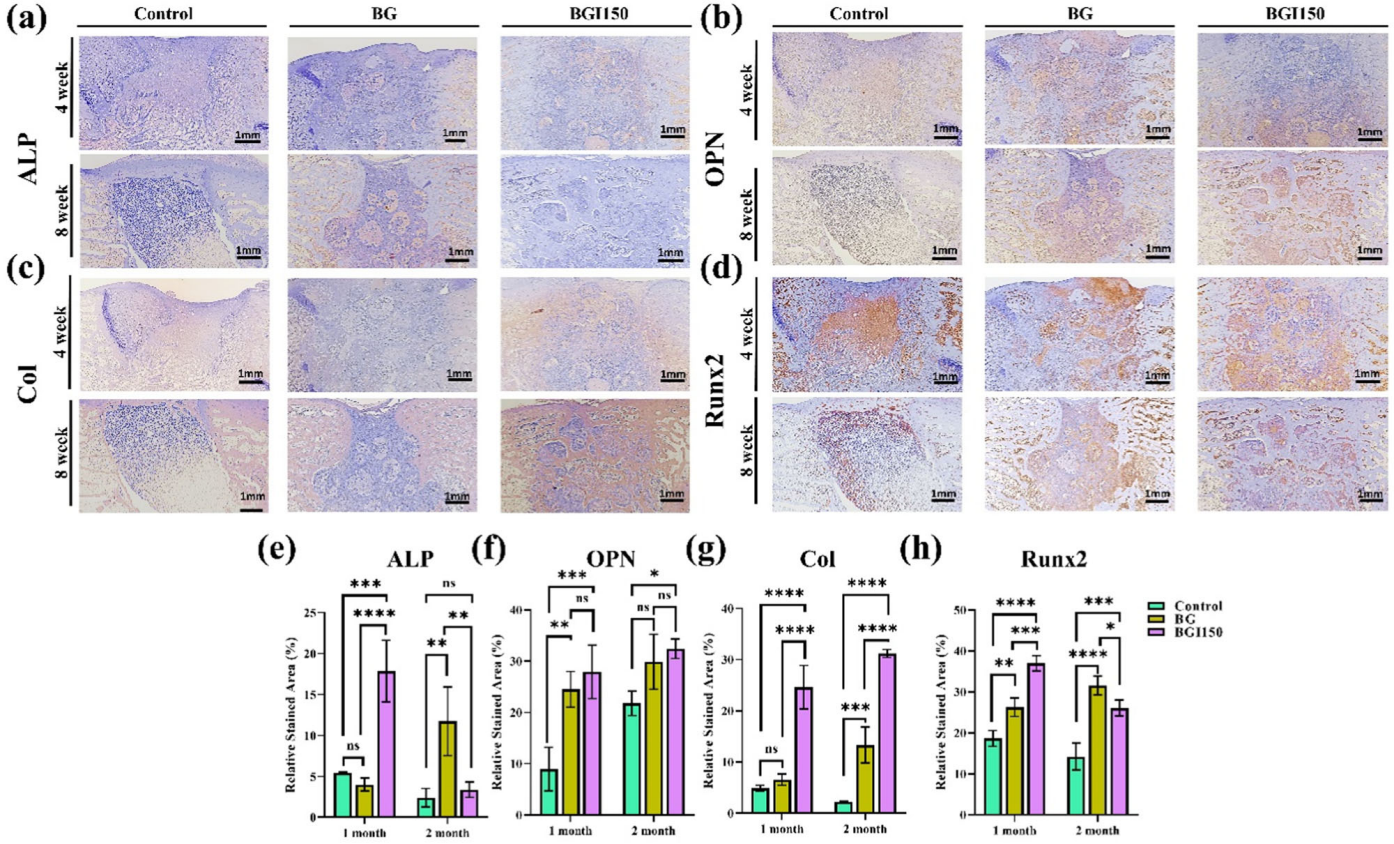
**a** ALP, **b** OPN, **c** Col, and **d** Runx2 antibody staining images for 4 and 8 weeks, and **e**–**h** representative quantification data. (****P < 0.0001, ***P < 0.001,**P < 0.01, *P < 0.05,and ns not significant; *n* = 3)

The in vivo osteogenic ability was evaluated by the immunohistochemistry analysis. We stained the defect site with the four markers, Runx-2, OPN, ALP, and Col-1 (Fig. 6a–d). Runx-2, OPN, and ALP are the osteogenic markers, whereas Col-1 is a structural protein. Our results showed that all the samples exhibited positive signals for the markers. Quantification was further performed, which helped evaluate the osteogenic ability of the samples quantitatively (Fig. 6e–h). The results showed that although the bioglass samples showed osteogenic ability, irisin-loaded samples demonstrated higher expression of the osteogenic markers. ALP helps in the early stages of the mineralization of the bone. The results indicate that in the case of irisin-loaded bioglass, ALP is expressed higher than the control and the only bioglass samples. After 8 weeks, a decrease in expression could be seen as in the irisin-loaded bioglass, and bone formation had passed the early stage of mineralization. In contrast, in the case of the bioglass, the bone formation was delayed. From the in vivo results, it can be concluded that the combination of bioglass with irisin remarkably enhanced the bioglass’s osteogenic and bone formation ability.

## Discussion

Bone regeneration is a complex process that can be accelerated by the release of ions such as phosphate and calcium [7]. The biocompatible material that has the ability to degrade with the passage of time along with the release of bone accelerating ion is desired for the bone regeneration process. Bioglass fits the merits of a biocompatible material with an appropriate degradation rate and ionic release to accelerate the bone regeneration process. For bone regeneration, the presence of pores is recommended as cells can penetrate the scaffold to regenerate. The presence of pores also ensures the diffusion of nutrients and fluids due to the in-growth capillaries [43]. The porosity of the bioglass can be controlled by the PMMA concentration. As the PMMA burns the pores are generated in the interconnected form. The number and size of the pores are controlled by the PMMA [36]. EDS and XRD analysis showed that bioglass is formed [16]. The characterization of the bioglass by the XRD supported that the bioglass was prepared in the pure form.

Bone injuries are difficult to deal with when society is aging as the regeneration ability of the body is decreased. In this scenario, the biomolecules that can accelerate the regeneration process can be the answer to this problem. Irisin is a myokine that can accelerate the osteogenesis process. PVA is a water-soluble polymer. It is proposed that by loading irisin using PVA, a slower release can be ensured as PVA can act to secure the irisin molecules on the surface of the porous bioglass. As it dissolves in water, irisin is released, slowing the protein release mechanism. As the beads come in contact with the body fluid, the polymer, being soluble in water, dissolves slowly, and as the polymer dissolves, irisin is also released [44]. The size of the pores also increases gradually as the polymer dissolves, thereby rendering the interconnected pores available for the proliferation of the cells. Drug release is crucial for the organism’s safety and effective therapeutics [45]. The overdose as a result of burst release can be harmful. Slow release can also delay the process of regeneration. Irisin is known to enhance osteogenic differentiation [46]. It was hypothesized that the bioglass beads loaded with irisin can effectively incline bone regeneration. Bioglass is a biodegradable material. The loading of PVA and irisin has not affected the rate of the degradation. The increase in the tensile strength and young modulus be can attributed to the PVA loading on the bioglass.

For the biocompatibility of the degradable materials, the measurement of the samples’ pH is crucial. In the case of bone regeneration, it is observed that elevation in the pH is known to help in proliferation and differentiation and increase mineralization in the pre-osteoblastic cells [47, 48]. For the fast regeneration of the bone accelerated osteoblast differentiation is necessary. The enhanced ability of the MC3T3E1 cells to deposit Ca can be directly related to osteoblast differentiation. As reported previously, slightly increased pH stimulates the glycolytic activity by calcium ion release [42].

The bioglass beads are highly porous, which helps the cells to proliferate, and with the release of ions, bone formation is also enhanced. When the bioglass samples are loaded with irisin, cell proliferation, and differentiation behavior is enhanced further. Hence, the irisin-loaded bioglass beads showed a higher ratio than the bioglass sample.

**Fig. 7:**
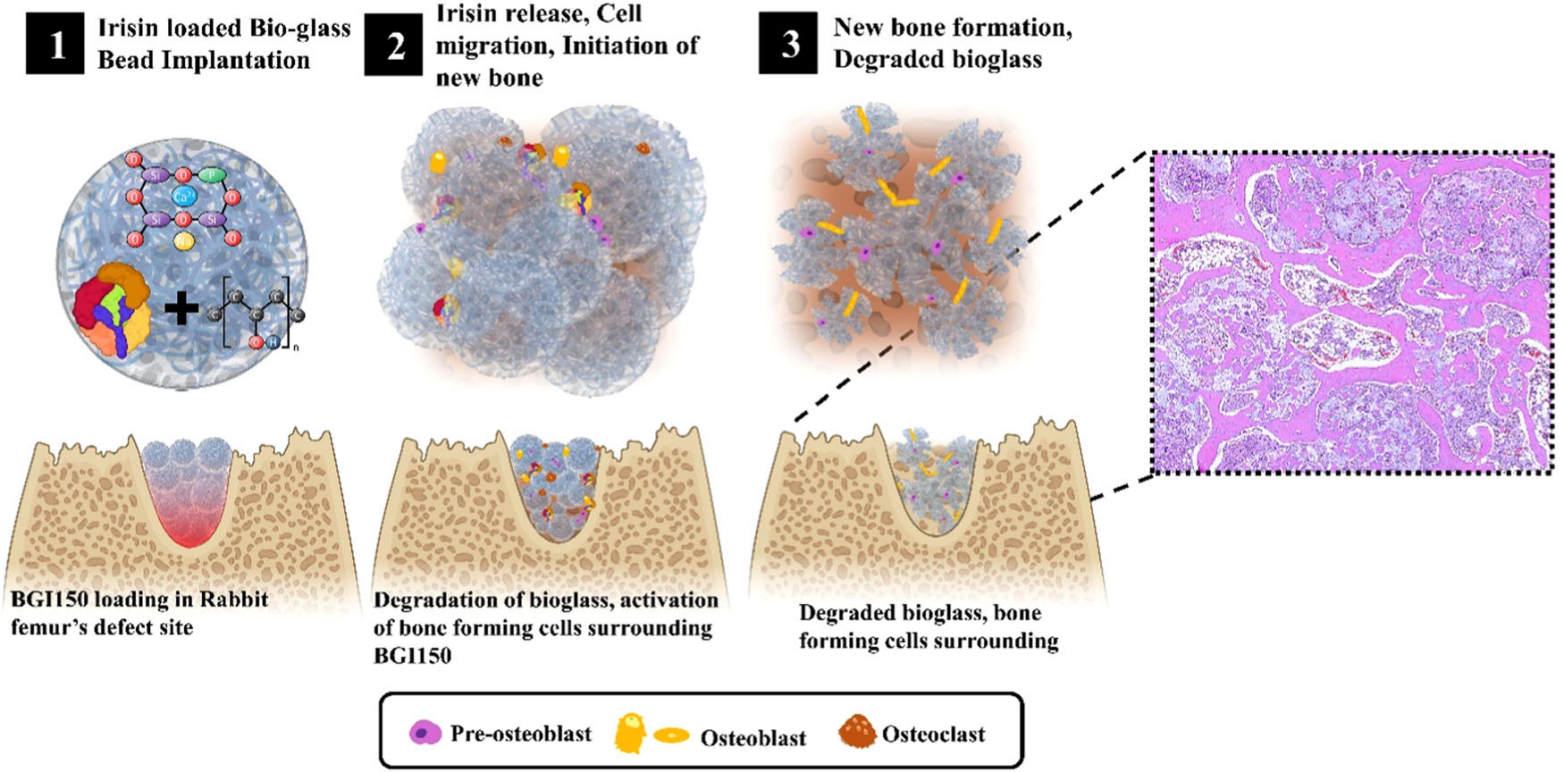
Graphical illustration representing the summary of bone regeneration by the irisin-loaded bioglass beads

Figure 7 is the summary illustration of this work. When the irisin-loaded bioglass is introduced in the defect site, the degradation of the bioglass starts, and irisin is released, resulting in vascularization, cell migration, and new bone formation. With the progression of time, degradation of bioglass occurs with the formation of new compact bone. The bone formation is evidently enhanced by the loading of the irisin on the porous bioglass.

## Conclusion

In this work, we prepared bioglass beads and loaded them with irisin and PVA. PVA was used as a binder material to sustain the release of irisin. The in vitro evaluation showed that bioglass and irisin-loaded samples were not biocompatible but also had osteogenic properties. To validate the in vitro results, in vivo experiments were carried out. In vivo, results showed that irisin loading on the bioglass beads enhanced bone formation in the rabbit femur compared to the bioglass sample. The histological data showed that bone formation could be observed between the irisin-loaded beads after 8 weeks, whereas only bone marrow was observed in the bioglass beads. Immunohistochemical analysis also supported the enhancement of bone formation due to irisin loading. It can be concluded that using irisin is a promising strategy for future bone-related problems.
